# Omental rhabdomyosarcoma (primary rhabdoid tumor of greater omentum): a rare case report

**DOI:** 10.1186/s40792-015-0077-6

**Published:** 2015-09-02

**Authors:** Priyank Pathak, Mayank Nautiyal, P. K. Sachan, Nadia Shirazi

**Affiliations:** Department of General Surgery, Himalayan Institute of Medical Sciences, Swami Rama Himalayan University (SRHU), Jollygrant, Doiwala, Dehradun 248140 India

**Keywords:** Rhabdomyosarcoma, Greater omentum

## Abstract

The greater omentum is an uncommon location for primary tumors. Metastatic tumors of the omentum are common. In contrast, primary tumors of the omentum are very rare. Sporadic cases of primary rhabdomyosarcoma (RMS) arising in the abdomen have been reported, but these cases are limited almost exclusively to the pediatric population. We report a well-documented case of primary intra-abdominal RMS in a 21-year-old man who presented with complaints of abdominal pain and lump in left hypochondrium region. Imaging revealed it to be a large mass in the left hypochondrium region displacing the bowel loops. Further investigations revealed omental RMS. The mass had originated from the greater omentum and was excised. Our case is doing well and is presently receiving chemotherapy.

## Background

RMS is an uncommon neoplasm in the adult population. The name is derived from the Greek words *rhabdo*, which means rod shape, and *myo*, which means muscle. Sporadic cases of primary RMS arising in the abdomen have been reported, but these cases are limited almost exclusively to the pediatric population. Intraperitoneal RMS, and in particular with omental involvement, in any age has been rarely reported in literature. RMS usually manifests as an expanding mass. Tumors in superficial locations may be palpable and detected relatively early, but those in deep locations (e.g., retroperitoneum) may grow large before causing symptoms. Cells are usually positive for intermediate filaments and other proteins typical of differentiated muscle cells, such as desmin, vimentine, myoglobin, actin, and transcription factor myoD. Treatment responses and prognoses widely vary depending on location and histology. While the optimal management of this rare tumor is unknown, early recognition and diagnosis, and a prompt multimodality treatment approach of surgery, chemotherapy, and radiotherapy offers the best chance of cure.

## Case report

**Fig. 1:**
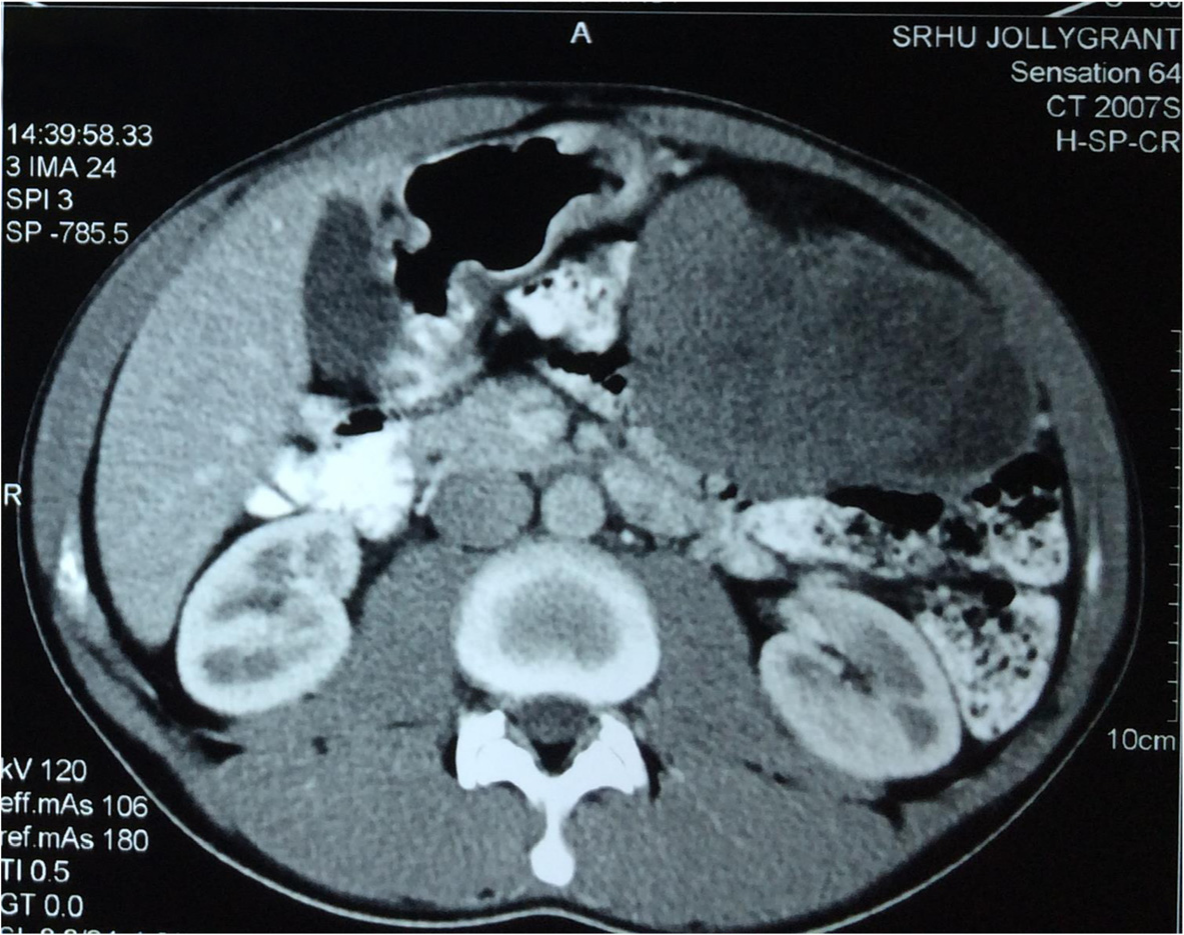
Showing a large well defined mass of 9.8 × 7.4 cms along with displacement of the bowel loops

A 21-year-old-man was admitted to our hospital with a 1-month history of abdominal pain. Physical examination revealed a palpable mobile lump in the left hypochondrium region and extending up to the epigastrium region. There is no past history of any surgical interventions and any chronic illness. No positive family history of any hereditary disease or any carcinoma. No abnormalities in routine blood workup. Ultrasound abdomen was suggestive of a well defined but irregular hypoechoic mass lesion in the left hypogastric region and extending into the lumbar region. Colonoscopy was done which was negative for any intramural growth and no signs of malignancy. Ultrasound guided FNAC from the left hypochondrium region showed deposits of adenocarcinoma. The abdominal CT revealed a large well defined mass lesion in the left hypochondrium, measuring 9.8 × 7.4 cms, causing displacement of the bowel loops (Fig. 1).

**Fig. 2:**
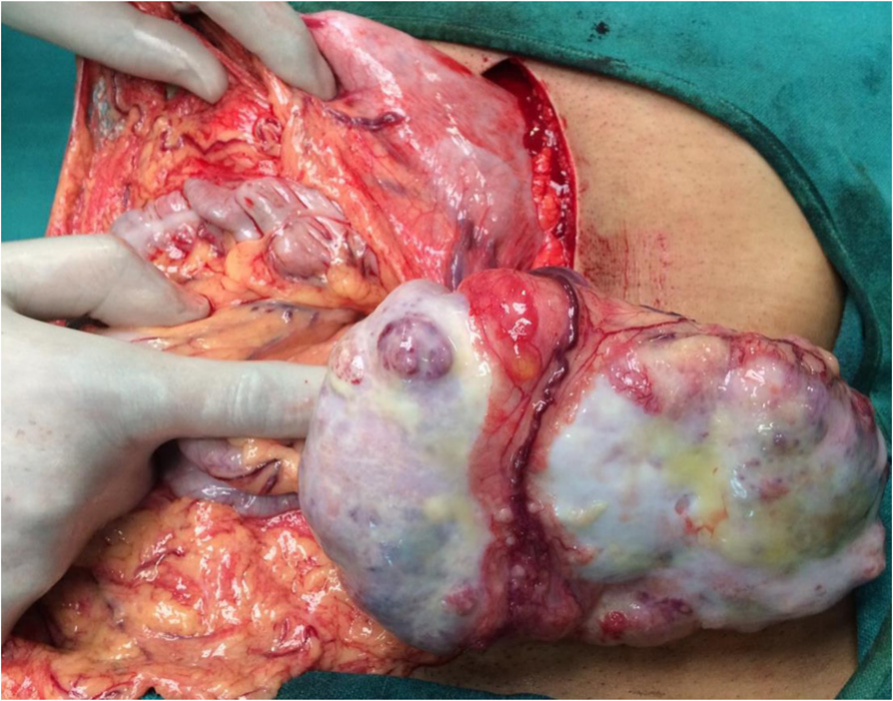
Intra operative finding of a large omental tumor in the left hypochondrium region with areas of ulceration

Intraoperative, a large hard, nodular necrotic mass encased within the greater omentum present at the splenic flexure. Some peri splenic and para aortic lymph nodes were present which were excised in block (Fig. 2).

**Fig. 3:**
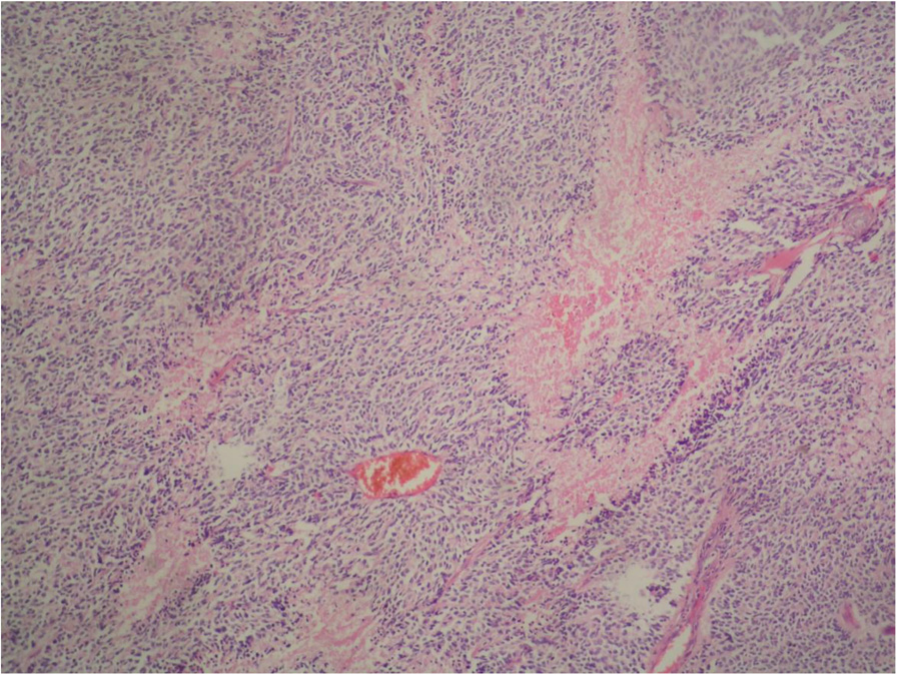
H and E; 10 × 10 X shows malignant round cell tumor arranged in sheets admixed with areas of necrosis

The histopathological diagnosis of the patient was reported as poorly differentiated malignant round cell tumor with metastasis in regional lymph nodes and perinodal extension. Lymph nodes were free of tumor, and there was only reactive hyperplasia. On cross section examination, cut surface was homogenous grayish white along with many areas of hemorrhage and necrosis. On microscopy, tumor cells have round to mildly pleomorphic nuclei, with coarse chromatin and indistinct cell borders. Large areas of tumor necrosis are seen (Fig. 3).

**Fig. 4:**
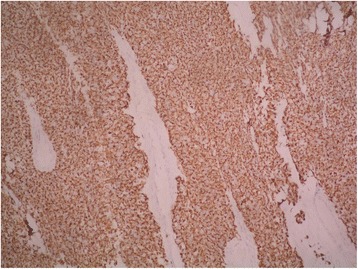
Immunohistochemistry, 10 × 10 X shows tumor cells showing desmin positivity

**Fig. 5:**
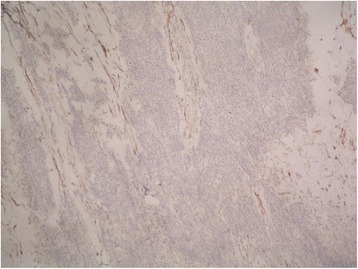
Immunohistochemistry 10 × 10 X shows tumor cells are negative for pancytokeratin

Immunohistochemistry was planned in view of multiple differential diagnoses in the histopathological report. Immunohistochemistry revealed strong staining for Vimentin, EMA, and desmin, thereby confirming the diagnosis for RMS (Figs. 4 and 5). The postoperative period was uneventful. Postoperatively, upper gastrointestinal endoscopy was done which was normal. He was discharged on the third postoperative day. Patient underwent PET scan, which turned out to be negative for any primary disease or other secondary deposits. Patient is undergoing chemotherapy (4 cycles). Patient is presently on vincristine, dactinomycin, and ifosfamide regimen (vincristine: 1.5 mg/m2 iv on day 1, dactinomycin 0.75 mg/m2 iv on day 1 and 2, mesna prior to ifosfamide 1gm/m2 on day 1 and 2, and then ifosfamide 1.5gm/m2 iv on day 1 and 2). Patient is doing well and has completed all the cycles of chemotherapy. Blood investigations done routinely are all within normal limits. Patient has been counseled to come for follow up every 6 months. We do not expect any alterations in the expected survival age of the patient.

## Discussion

Contrary to metastatic tumors of the omentum, primary tumors of the omentum are very rare and sporadically reported. However, a primary malignant rhabdoid tumor in the omentum is very rare. RMS rarely affects adults. AS per the Pubmed survey, only three cases have been reported yet. The rhabdoid cells show round to tear drop shape with vesicular nuclei and a single large nucleolus. There are ill-defined round to oval hyaline inclusions composed of intermediate filaments in cytoplasm [1].

Intraperitoneal RMS, and in particular with omental involvement, in any age has been rarely reported in literature [2]. In general, the symptoms of omental tumors present as abdominal discomfort (45.5 %), abdominal mass (34.9 %), and abdominal distention (15.2 %). Unfortunately, there are no specific findings differentiating the origin or nature of the mass in imaging studies due to the extent of the omentum and the adhered organs. A considerable finding is displacement of the stomach, the transverse colon, and small bowel, by an extrinsic mass [3].

The treatment of omental tumors is complete excision via omentectomy. We diagnosed the mass as a malignant rhabdoid tumor based on the typical cellular morphology and immunohistochemical stains. Immunohistochemically, the rhabdoid cells express both cytokeratin and vimentin, but not myogenic differentiation nor INI1 protein [4].

For treatment, an aggressive operation to achieve total resection is recommended because the effectiveness of chemo radiotherapy has not been proven. The classic combination of ifosfamide, carboplatinum, and etoposide is recommended, and multidrug protocols including topoisomerase I inhibitors are recommended [5].

Vincristine, actinomycin, and cyclophosphamide (VAC) compared with vincristine, Actinomycin, and Cyclophosphamide with Vincristine, topotecan, and cyclophosphamide (VAC/VTC) for intermediate rhabdomyosarcoma was done at weeks 3, 9, 21, 27, 33, 39 did not show an improvement in outcome for patients with intermediate-risk RMS. The estimated 4-year FFS rates were 73 % for VAC and 68 % for VAC/VTC, which did not differ significantly [6].

Radiotherapy plays an important role in the treatment of RMS and is a major tool in the treatment of children. A cumulative dose of between 36 and 41.4 Gy is generally sufficient to control microscopic residual disease, and doses of between 50.4 and 54 Gy are utilized to control gross residual disease. Patient is to be followed every 3 months for the 1st year, then every 6 months for the 2nd and 3rd year and then yearly after [7].

## Conclusions

The diagnosis of tumors in the omentum is very difficult and requires confirmation by excision and histologic evaluation. A primary tumor originating in the omentum is rare but possible even as a malignant tumor. The prognosis depends mainly on tumor biology yet the malignancy of the omentum might cause severe outcomes because these masses can easily metastasize to mesentery and peritoneum tissues.

## Consent

Written informed consent was obtained from the patient for publication of this Case report and any accompanying images. A copy of the written consent is available for review by the Editor-in-Chief of this journal.

## Abbreviations

RMS: rhabdomyosarcoma
